# Immunogenicity and safety of two monovalent rotavirus vaccines, ROTAVAC® and ROTAVAC 5D® in Zambian infants

**DOI:** 10.1016/j.vaccine.2021.04.060

**Published:** 2021-06-16

**Authors:** R. Chilengi, K. Mwila- Kazimbaya, M. Chirwa, N. Sukwa, C. Chipeta, R.M. Velu, N. Katanekwa, S. Babji, G. Kang, M.M. McNeal, N. Meyer, G. Gompana, S. Hazra, Y. Tang, J. Flores, N. Bhat, N. Rathi

**Affiliations:** a Centre for Infectious Disease Research in Zambia, Zambia; b The Wellcome Trust Research Laboratory, Vellore, India; c Department of Pediatrics, University of Cincinnati College of Medicine, Division of Infectious Diseases, Cincinnati Children’s Hospital Medical Center, Cincinnati, OH, USA; d PATH, India; e PATH, USA

**Keywords:** Rotavirus Vaccine, ROTAVAC, ROTAVAC 5D, Safety, Immunogenicity, Zambia

## Abstract

**Background and Aims:**

ROTAVAC® (frozen formulation stored at −20 °C) and ROTAVAC 5D® (liquid formulation stable at 2–8 °C) are rotavirus vaccines derived from the 116E human neonatal rotavirus strain, developed and licensed in India. This study evaluated and compared the safety and immunogenicity of these vaccines in an infant population in Zambia.

**Methods:**

We conducted a phase 2b, open-label, randomized, controlled trial wherein 450 infants 6 to 8 weeks of age were randomized equally to receive three doses of ROTAVAC or ROTAVAC 5D, or two doses of ROTARIX®. Study vaccines were administered concomitantly with routine immunizations. Blood samples were collected pre-vaccination and 28 days after the last dose. Serum anti-rotavirus IgA antibodies were measured by ELISA, with WC3 and 89–12 rotavirus strains as viral lysates in the assays. The primary analysis was to assess non-inferiority of ROTAVAC 5D to ROTAVAC in terms of the geometric mean concentration (GMC) of serum IgA (WC3) antibodies. Seroresponse and seropositivity were also determined. Safety was evaluated as occurrence of immediate, solicited, unsolicited, and serious adverse events after each dose.

**Results:**

The study evaluated 388 infants in the per-protocol population. All three vaccines were well tolerated and immunogenic. The post-vaccination GMCs were 14.0 U/mL (95% CI: 10.4, 18.8) and 18.1 U/mL (95% CI: 13.7, 24.0) for the ROTAVAC and ROTAVAC 5D groups, respectively, yielding a ratio of 1.3 (95% CI: 0.9, 1.9), thus meeting the pre-set non-inferiority criteria. Solicited and unsolicited adverse events were similar across all study arms. No death or intussusception case was reported during study period.

**Conclusions:**

Among Zambian infants, both ROTAVAC and ROTAVAC 5D were well tolerated and the immunogenicity of ROTAVAC 5D was non-inferior to that of ROTAVAC. These results are consistent with those observed in licensure trials in India and support use of these vaccines across wider geographical areas.

## 1 Introduction

Rotavirus is the most common cause of severe diarrhea and is estimated to be responsible for 258 million episodes of diarrhea and 128 500 deaths (95% UI: 104 500–155 600) among children < 5 years of age globally. Of these, approximately 104 733 deaths (95% UI: 83 406–128 842) occurred in sub-Saharan Africa in 2016 [1], [2]. Four oral rotavirus vaccines, ROTARIX®, RotaTeq®, ROTAVAC® and ROTASIIL®, have been prequalified by the World Health Organization (WHO).

ROTAVAC was developed by Bharat Biotech International Limited (BBIL) in Hyderabad, India, and was licensed in 2014 based on satisfactory safety and efficacy studies conducted in India [3], [4], [5]. The initial formulation of ROTAVAC was a 3-dose vaccine administered with a 2.5 mL citrate-bicarbonate buffer to facilitate passage through the acidic contents of the upper gastrointestinal tract. Further evaluation led to the demonstration of similar immune responses by ROTAVAC when administered with and without antacid buffer [6]. ROTAVAC can be administered concomitantly with routine childhood vaccines at 6, 10, and 14 weeks of age [7] and has demonstrated non-inferior immunogenicity when compared to ROTARIX in studies conducted in India [8]. Although this vaccine has a low dose volume, is suitable for infant use, and does not require an alkali buffer, it still requires freezer storage at −20 °C, which can be an impediment for countries lacking freezer storage. ROTAVAC received WHO pre-qualification in 2018 and has received licensure in several African countries.

In order to make ROTAVAC more heat-stable BBIL developed a new formulation, ROTAVAC 5D**®**. This formulation has additional excipients and stabilizers that belong to the Generally Regarded As Safe (GRAS) category and thus no new safety concerns were anticipated. It has a recommended volume of 0.5 mL (approximately 5 drops) and is stable at 2–8 °C for over 24 months. This formulation contains the same rotavirus strain (116E) as ROTAVAC and elicits non-inferior immunogenicity among Indian infants (data under publication). ROTAVAC 5D also has exhibited stability at 25 °C for 8 weeks and at 37 °C for 1 week(data on file with the manufacturer).

The present study in Zambian infants was undertaken to evaluate the immunogenicity of ROTAVAC 5D in comparison to ROTAVAC, and to assess the safety and reactogenicity of these two formulations in a population that is outside of India and bears a high burden of rotavirus disease. Recent data from Zambia have recorded significant reductions in acute gastroenteristis-associated in-hospital morbidity and mortality following vaccine introduction. The greatest reduction was noted in infants, with the rotavirus positivity rate in this age group declining from 40.9% in pre-vaccine years to 34.0% (P = .009) in 2013 and 26.2% (P < .001) in 2014. This residual burden needs to be pushed back until all preventable hospitalizations are averted. A part of this problem is a failure of current vaccines to completely prevent infections/disease in vaccinated infants possibly because of low effectiveness [9]. Therefore new and alternative vaccines are presently needed, and this work is intended to provide evidence of ROTAVAC performance across a broader geography to support informed policy decision-making regarding rotavirus vaccine product choice by global, national, and regional immunization authorities.

## 2 Methods

### 2.1 Ethics

The study was reviewed and approved by the University of Zambia Biomedical Research Ethics Committee (UNZABREC Ref: 021–07-18) and by Western Institutional Review Board (WIRB Ref: 20182158). The study was also approved by the Zambia Medicines Regulatory Authority (ZAMRA Ref: CT080/18)) and the Zambian National Health Research Authority (NHRA) prior to study initiation. The study conduct complied with the Declaration of Helsinki and Good Clinical Practice guidelines and is registered at ClinicalTrials.gov as NCT 03602053.

### 2.2 Study design

The Phase IIb, single-center, randomized, active-controlled, open-label study was conducted between January 2019 and October 2019 at George Health Centre in Lusaka, Zambia. Families of infants attending the centre for routine vaccinations at 6 weeks were approached and those interested were screened after obtaining written consent. Eligible participants were equally randomized using masked allocation to receive either ROTAVAC, ROTAVAC 5D, or ROTARIX. ROTARIX was included as a control arm as it is used for routine immunization of children in Zambia. Three doses of ROTAVAC and ROTAVAC 5D were administered at 6, 10, and 14 weeks of age whereas two doses of ROTARIX were administered at 6 and 10 weeks of age. All study participants concomitantly received other Expanded Program on Immunization (EPI) vaccines including the combined pentavalent vaccine (containing diphtheria, tetanus, whole cell pertussis, haemophilus influenza type b, and hepatitis B), pneumococcal conjugate vaccine, and oral polio vaccine.

### 2.3 Selection criteria

Participants were healthy infants between the age of 6 and 8 weeks and whose parents were willing to provide written consent and agreed to be followed up. Potential participants with impaired immunological function, those who were administered immunoglobulin therapy, blood products, or immunosuppressants, or those with severe malnutrition, neurologic disorders, significant congenital anomaly, or allergy to any component of the study vaccine were excluded from the study. Other important exclusion criteria were a history of congenital abdominal disorders, intussusception, abdominal surgery, or persistent diarrhea and those who preferred to receive rotavirus vaccine from outside the study center.

### 2.4 Randomization and blinding

Eligible participants were randomized 1:1:1 to receive either ROTAVAC, ROTAVAC 5D, or ROTARIX. Block randomization with a block size of 6 was used and the prospective treatment schedules were masked to avoid any selection bias in treatment allocation. Due to the differing dosing schedules among the arms, the parents and study staff were unblinded and aware of the treatment allocations after randomization. However, all immunological testing at both laboratories was conducted under blinded conditions.

### 2.5 Investigational products

ROTAVAC and ROTAVAC 5D are live attenuated monovalent vaccines containing not less than (NLT) 10^5.0^ focus forming units (FFU) per dose of 116E (G9P [11]) rotavirus strain manufactured by BBIL, Hyderabad, India. ROTAVAC is stored at −20 ˚C but can be maintained at 2-8˚C for a maximum of 8 h on the day of use. It is administered orally in a dose of 0.5 mL (5 drops) at 6, 10, and 14 weeks of age. Although, ROTAVAC was supplied in a 10-dose vial, only one dose per child per vial was used in the study and the remainder was discarded. The batch numbers of ROTAVAC used in the study were 61FA16021 and 61C18040A. ROTAVAC 5D is stored and transported at 2-8˚C and requires no thawing. It is administered orally in a dose of 0.5 mL (5 drops) at 6, 10, and 14 weeks of age. The batch number of ROTAVAC 5D used in the study was 61GD17002.

ROTARIX is a ready-to-use, live attenuated G1P[8] monovalent vaccine containing NLT 10^6.0^ CCID50 (cell culture infectious dose 50%). The vaccine is manufactured by GlaxoSmithKline Biologicals, Rixensart, Belgium. It is administered orally in a dose of 1.5 mL at 6 and 10 weeks of age and is stored and transported at 2-8˚C. The batch number of ROTARIX used in the study was AROLCO54AA.

All three study vaccines did not require reconstitution or dilution, and were administered immediately after the vial was opened.

## 3 Outcomes

### 3.1 Immunogenicity assessment

Blood samples were obtained from all the participating infants before the first vaccination and four weeks after the last vaccine dose; i.e., at approximately 14 weeks of age for infants in the ROTARIX arm and 18 weeks of age for infants in both ROTAVAC arms. Samples were allowed to clot for at least 30 min and were centrifuged at 2500 rpm and the serum aliquoted, labelled, and stored at −80 °C.

All samples were tested for serum anti-rotavirus immunoglobulin A (IgA) antibodies by a validated ELISA using WC3 virus as the antigen at the Wellcome Trust Research Laboratory, Christian Medical College (CMC), Vellore, India. A subset of samples (50 sample pairs per arm) was also analysed by another ELISA using the ROTARIX-specific strain 89–12 as the antigen. This testing was performed at the Laboratory for Specialized Clinical Studies, Cincinnati Children's Hospital Medical Centre (CCHMC), Cincinnati, Ohio, USA. Both laboratories used an assay developed in conformance with the WHO *Manual of Rotavirus Detection and Characterization Methods*[10], [11], [12], [13]. The assays had acceptable accuracy, precision, and linearity at both testing laboratories, with a standard curve modelled using a 4-parameter logistic fit regression function. During validation of the assay, the lower limit of quantification was set at 7.0 U/mL and 7.5 U/mL at CMC and CCHMC, respectively.

The primary endpoint for immunogenicity was GMC of serum anti-rotavirus IgA (WC3) antibodies. Other endpoints included seropositivity, seroresponse, and seroconversion. Seropositivity was defined as anti-rotavirus IgA concentration ≥ 20 U/ml. Seroresponse was defined as a post-vaccination IgA antibody concentration of ≥ 20 U/mL and 4-fold baseline level if a baseline concentration is greater than the lower limit of quantification (LLOQ), or a post-vaccination IgA antibody concentration of ≥ 20 U/mL and 4-fold LLOQ if a baseline concentration is less than or equal to the LLOQ. Seroconversion was defined as a post-vaccination IgA antibody concentration of at least 20 U/mL if a baseline concentration is < 20 U/mL or a post-vaccination serum anti-rotavirus IgA antibody concentration of ≥ 2-fold baseline level if a baseline concentration is ≥ 20 U/mL.

### 3.2 Safety assessment

All participants were monitored for 30 min for immediate adverse events (AEs). Enhanced passive/active surveillance for vaccine reactogenicity (solicited reactions of fever, diarrhea, vomiting, decreased appetite, irritability, decreased activity level) over the 7-day period after each vaccination was conducted on all infants. Parents used a digital thermometer and a Post-Immunization Diary Card (PIDC) to record the presence or absence of a solicited AE, its severity, and the use of concomitant medication. The staff visited the participants’ homes twice during the 7-day period to determine the health status of the child and support completion of the PIDC. Unsolicited AEs were reported spontaneously by the participant, observed by the study personnel during study visits or identified during review of medical records or source documents. Unsolicited AEs and serious AEs were monitored during the period from first vaccination through 4 weeks after the last vaccination. The overall follow-up period was shorter for the Rotarix arm as only 2 doses were administered. The severity of the events was graded by the investigator based on guidance in the protocol.

A Protocol Safety Review Team (PSRT) comprised of physicians from within the study team and an independent physician met periodically to review the study data.

### 3.3 Statistical considerations

The full analysis (FA) population included all enrolled participants who were randomized and received at least one dose of study vaccination and provided at least one evaluable serum sample. The per-protocol (PP) population included all participants in the FA population who had correctly received study vaccine per randomization with no protocol violations that was determined to potentially interfere with the immunogenicity assessment of the study vaccines. The PP population was the primary analysis population for all immunogenicity analyses, while the FA population results were supportive. The safety population, which was used for all safety analyses, included all enrolled participants who received a study vaccination and had any safety data available. One-sided type I error rate of 0.025 was used for the non-inferiority comparisons. All statistical analyses were conducted using SAS® software, version 9.4 (SAS Institute Inc., Cary, NC, USA).

Assuming the true standard deviation of log_10_-transformed anti-rotavirus IgA concentration is below or equal to 0.60 and allowing a dropout rate of 10%, a sample size of 150 participants per arm provided 98% power to detect non-inferiority of ROTAVAC 5D to ROTAVAC in terms of GMC of serum anti-rotavirus IgA (WC3) antibodies. The non-inferiority margin was 0.5.

### 3.4 Immunogenicity analysis

For the primary immunogenicity analysis, the GMCs of serum anti-rotavirus IgA (WC3) antibodies at 28 days after the last dose of ROTAVAC and ROTAVAC 5D were calculated along with their two-sided 95% confidence intervals (CI), by exponentiating the corresponding log_10_-transfomed mean and its two-sided 95% CI limits. To compare the immunogenicity of ROTAVAC and ROTAVAC 5D, the ratio of the post-vaccination GMCs between ROTAVAC 5D and ROTAVAC study groups was calculated with a two-sided 95% CI. The log_10_-transformed IgA (WC3) concentrations were used to construct a two-sided 95% CI for the mean difference between the two study groups using the t-distribution. The mean difference and corresponding 95% CI limits were exponentiated to obtain the GMC ratio and the corresponding 95% CI. The *a priori* condition was that if the lower limit of the 95% CI of the ratio of GMCs between the ROTAVAC 5D and ROTAVAC groups was>0.5 (non-inferiority margin), ROTAVAC 5D would be considered non-inferior to ROTAVAC. A supportive comparison of the GMCs between the two study vaccines was also performed using Analysis of Covariance (ANCOVA) to adjust for baseline concentration.

The percentage of participants with seroconversion and seroresponse were also assessed for the two ROTAVAC groups along with exact two-sided 95% CI computed by the Clopper-Pearson method. The difference in the percentage between the two groups was calculated along with its two-sided 95% CI obtained by the Miettinen and Nurminen method [14].

The geometric mean fold ratio (GMFR), defined as GMC post-vaccination divided by GMC at baseline, was calculated with its two-sided 95% CIs by exponentiating the difference in means of log_10_-transformed anti-rotavirus IgA concentrations between post-vaccination and baseline. The two-sided 95% CIs were calculated using the paired *t*-test. Reverse cumulative distribution curves were generated. The immunogenicity analysis for ROTARIX and the exploratory analysis conducted on IgA responses using 89–12 (G1P[8] virus) as the antigen (viral lysate) were descriptive.

### 3.5 Safety analysis:

Adverse events (AEs) were classified and tabulated as immediate AEs (IAEs), solicited AEs, unsolicited AEs, and serious AEs (SAEs) and categorized by severity and causality. Proportions of these safety endpoints along with their exact two-sided 95% CI were provided.

## 4 Results

**Fig. 1:**
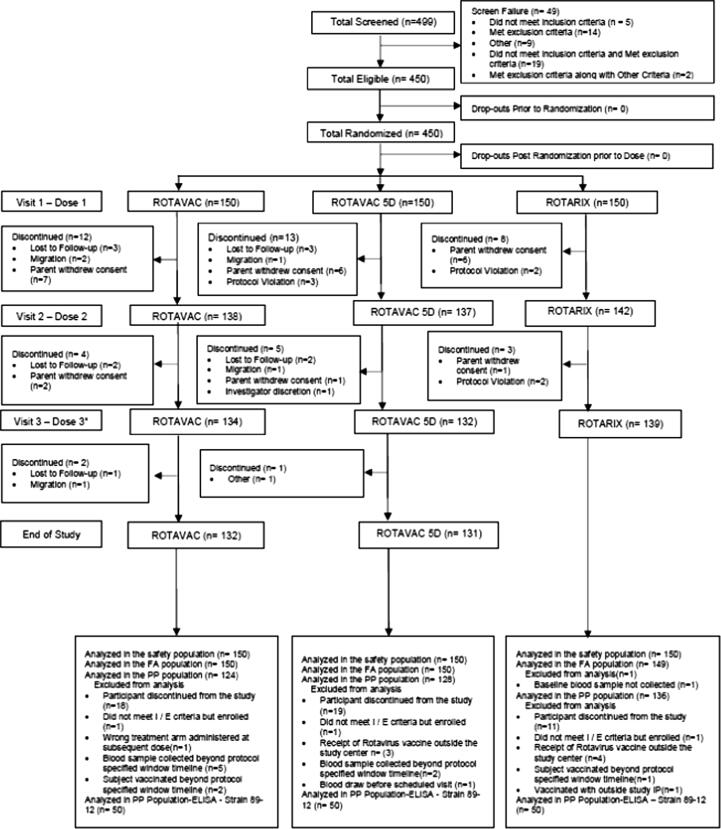
Study Flow Chart.

A total of 499 participants were screened. Of these, 450 participants, distributed almost equally between males and females, were found eligible and randomized to one of the three study groups. All enrolled participants were Black Africans aged between 5.9 and 8 weeks of age with a mean weight 4.8 kg (range 3.3–6.9). There were no differences in the baseline demographic characteristics by study arm as summarized in Table 1. A total of 132 (88.0%) participants in the ROTAVAC arm, 131 (87.3%) in the ROTAVAC 5D arm, and 139 (92.7%) in the ROTARIX arm completed the study. As summarized in Figure 1, 62 participants were excluded from the per-protocol population because of either participant discontinuation or one or more protocol deviation affecting immune response. Therefore, the PP population included a total of 388 participants with a distribution of 124 (82.7%), 128 (85.3%), and 136 (90.7%) in the ROTAVAC, ROTAVAC 5D, and ROTARIX arms, respectively. Data on vaccine exposure are described in Fig. 1.

**Table 1:** Summary of Baseline Characteristics: Demographics and Other Characteristics- Enrolled Population.

Demographics Characteristic	Statistic	Rotavac (N = 150)	Rotavac 5D (N = 150)	Rotarix (N = 150)	Total (N = 450)	p-value
**Gender**						0.3537
Male	n (%)	73 (48.7%)	84 (56.0%)	84 (56.0%)	241 (53.6%)	
Female	n (%)	77 (51.3%)	66 (44.0%)	66 (44.0%)	209 (46.4%)	
**Race**						–
Black African	n (%)	150 (100.0%)	150 (100.0%)	150 (100.0%)	450 (100.0%)	
Mixed Race (Coloured)	n (%)	0	0	0	0	
Other	n (%)	0	0	0	0	
**Ethnicity**						0.4868
Bemba	n (%)	48 (32.0%)	60 (40.0%)	52 (34.7%)	160 (35.6%)	
Tonga	n (%)	10 (6.7%)	10 (6.7%)	6 (4.0%)	26 (5.8%)	
Chewa	n (%)	31 (20.7%)	30 (20.0%)	26 (17.3%)	87 (19.3%)	
Others	n (%)	61 (40.7%)	50 (33.3%)	66 (44.0%)	177 (39.3%)	
**Age at Baseline (Week)**	n (Missing)	150 (0)	150 (0)	150 (0)	450 (0)	0.1894
	Mean (SD)	6.5 (0.5)	6.6 (0.5)	6.6 (0.5)	6.6 (0.5)	
	Median	6.4	6.4	6.6	6.4	
	(Min, Max)	(5.9, 8.0)	(5.9, 8.0)	(5.9, 8.0)	(5.9, 8.0)	
**Birth Weight (Kg)**	n (Missing)	150 (0)	150 (0)	150 (0)	450 (0)	0.6290
	Mean (SD)	3.1 (0.5)	3.1 (0.4)	3.1 (0.4)	3.1 (0.4)	
	Median	3.0	3.1	3.1	3.1	
	(Min, Max)	(2, 4.7)	(2, 5)	(2, 4.1)	(2, 5)	
**Weight at Baseline (Kg)**	n (Missing)	150 (0)	150 (0)	150 (0)	450 (0)	NA
	Mean (SD)	4.8 (0.6)	4.9 (0.6)	4.9 (0.6)	4.8 (0.6)	
	Median	4.8	4.8	4.9	4.8	
	(Min, Max)	(3.3, 6.9)	(3.4, 6.5)	(3.5, 6.5)	(3.3, 6.9)	
**Length at Baseline (cm)**	n (Missing)	150 (0)	150 (0)	150 (0)	450 (0)	NA
	Mean (SD)	54.0 (2.0)	54.2 (2.3)	54.3 (2.1)	54.2 (2.1)	
	Median	54.0	54.0	54.0	54.0	
	(Min, Max)	(49.0, 60.0)	(47.0, 61.0)	(49.0, 59.0)	(47.0, 61.0)	
NA: Not Assessed.

### 4.1 Immunogenicity of ROTAVAC and ROTAVAC 5D

Baseline pre-vaccination anti-rotavirus IgA GMCs were in the range of 3.8–3.9 U/mL and were similar in the two ROTAVAC groups (pre-vaccination GMC ratio being 1.0 (95% CI: 0.9, 1.1)). Anti-rotavirus IgA GMCs at 28 days post dose 3 were also comparable between ROTAVAC 5D and ROTAVAC (GMC: 18.1 (95% CI: 13.7, 24.0) versus 14.0 (95% CI: 10.4, 18.8), respectively), and their GMC ratio (ROTAVAC 5D/ ROTAVAC) was 1.3 (95% CI: 0.9, 1.9) with the lower limit of the 95% CI being > 0.5. This supports the non-inferiority of the immunogenicity of ROTAVAC 5D to that of ROTAVAC. This result was corroborated by ANCOVA analysis, which adjusted for the effects of baseline IgA levels (Table 2)

**Table 2:** Post- Vaccination Immune Responses for ROTAVAC and ROTAVAC 5D, in terms of Serum Anti-Rotavirus IgA Antibody Concentrations Measured by ELISA with WC3 Rotavirus Strain as Viral Lysate– PP Population.

	Rotavac			Rotavac 5D			Comparison
	N	n	GMC/ % / GMFR (95% CI)	N	n	GMC/ % / GMFR (95% CI)	GMC Ratio (Rotavac 5D/ Rotavac)/ % difference / GMFR Ratio (Post-vaccination/ Pre-vaccination) (95% CI)
GMC- *t*-test	124	124	14.0 (10.4, 18.8)	128	128	18.1 (13.7, 24.0)	1.3 (0.9, 1.9)
GMC –ANCOVA	124	124	14.0 (10.5, 18.8)	128	128	18.0 (13.6, 24.0)	1.3 (0.9, 1.9)
Seropositivity Rate	124	42	33.9 (25.6, 42.9)	128	54	42.2 (33.5, 51.2)	8.3 (-3.7, 20.1)
Seroconversion rate	124	41	33.1 (24.9, 42.1)	128	52	40.6 (32.0, 49.7)	7.6 (-4.4, 19.3)
Seroresponse rate	124	34	27.4 (19.8, 36.2)	128	43	33.6 (25.5, 42.5)	6.2 (-5.2, 17.4)
Geometric Mean Fold Rise (GMFR)	124	124	3.6 (2.7, 4.9)	128	128	4.6 (3.5, 6.1)	1.3 (0.8, 1.9)
Note: N: Number of participants in PP Population, n: Number of participants contributing to the analysis or meeting the criterion.							

The seropositivity rate (Serum rotavirus IgA > 20U/mL) was low at baseline, with only 1 (0.8%) and 4 (3.1%) participants in the ROTAVAC and ROTAVAC 5D groups, respectively. Post-vaccination seropositivity was seen in 33.9% and 42.2% of the participants in the ROTAVAC and ROTAVAC 5D groups, respectively.

**Fig. 2:**
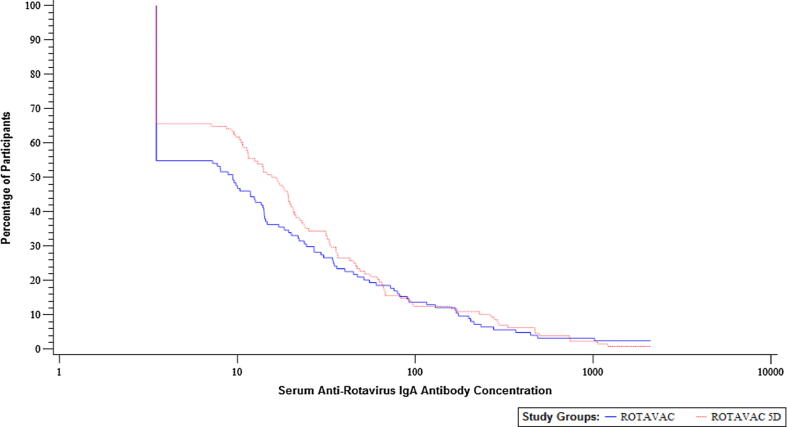
Reverse Cumulative Distribution Curves for IgA Antibody Immune Response Measured by ELISA using WC3 Viral Lysate at 28 Days after Last Dose for the Comparison of ROTAVAC with ROTAVAC 5D – PP Population.

Seroresponses in terms of a 4-fold response were calculated, with 27.4% and 33.6% of participants showing a 4-fold response in the ROTAVAC and ROTAVAC 5D groups, respectively. Given that the baseline seropositivity rates were low, the seroconversion rates were similar to seropositivity rates in the two groups at 33.1% and 40.6% in the ROTAVAC and ROTAVAC 5D groups, respectively. The GMFRs observed in the study groups were 3.6 (95% CI: 2.7, 4.9) and 4.6 (95% CI: 3.5, 6.1), respectively. Generally, the immune responses were similar in the ROTAVAC and ROTAVAC 5D groups, with the two-sided 95% CIs of their treatment group differences being inclusive of zero for proportions and inclusive of one for ratios. Seroresponse in terms of 2-fold and 3-fold rise in antibody levels were also calculated (refer to Supplementary Table 1). Reverse cumulative distribution curves are presented as Fig. 2.

### 4.2 Immune response to ROTARIX

The immunogenicity of ROTARIX was evaluated by ELISA using the fully homologous 89–12 rotavirus strain (source of the vaccine). Baseline pre-vaccination anti-rotavirus IgA GMC was 4.3 (95% CI: 3.7, 5.1) and post-vaccination GMC was 38.0 (95% CI: 20.5, 70.6). Other immunogenicity results included: post- vaccination seropositivity rate of 54.0% (95% CI: 39.3, 68.2); seroresponse in terms of a 4 -fold rise at 50.0% (95% CI: 35.5, 64.5); seroconversion rate of 52.0 (95% CI: 37.4, 66.3) and GMFR of 8.8 (95% CI: 4.7, 16.4).

### 4.3 Safety:

The safety population was comprised of 450 participants (150 in each group). Among them, two (0.4%) participants (one each in the ROTAVAC 5D and ROTARIX groups) reported pyrexia as an immediate adverse event (IAE). Both cases were mild, transient, and assessed as related to the study vaccine.

Solicited adverse reactions over the seven days post-vaccination were reported on 403 (92.4%) participants across all treatment groups, with 136 (94.4%) being in the ROTAVAC group, 138 (94.5%) in the ROTAVAC 5D group, and 129 (88.4%) in the ROTARIX group. The rate of solicited AEs and events following each dose were comparable between the two ROTAVAC groups. Fever and irritability were the most common, followed by decreased activity level, decreased appetite, vomiting and diarrhea as summarized in Table 3. All the participants had also received routine vaccinations, including DTwP-HepB-Hib vaccine which is known to be highly reactogenic. Nonetheless, the majority of the solicited AEs (1531 out of 1656 AEs) were of mild to moderate intensity and most had onset within one day of vaccination and resolved within 7 days of vaccination. All solicited AEs recovered, the majority (1429 of 1656) not necessitating any action such as medication, clinic visit, or hospitalization.

**Table 3:** Summary of Solicited Adverse Events by Maximum Severity – All Doses Combined – Safety Population.

Solicited AE	Rotavac (N = 144)		Rotavac 5D (N = 146)		Rotarix (N = 146)		Total (N = 436)	
	n (%), E	95% CI	n (%), E	95% CI	n (%), E	95% CI	n (%), E	95% CI
Any Solicited AEs	136 (94.4%), 604	(89.3, 97.6)	138 (94.5%), 615	(89.5, 97.6)	129 (88.4%), 437	(82.0, 93.1)	403 (92.4%), 1656	(89.5, 94.7)
Fever	109 (75.7%), 192	(67.9, 82.4)	110 (75.3%), 207	(67.5, 82.1)	100 (68.5%), 138	(60.3, 75.9)	319 (73.2%), 537	(68.7, 77.3)
Diarrhoea	11 (7.6%), 12	(3.9, 13.3)	15 (10.3%), 17	(5.9, 16.4)	8 (5.5%), 8	(2.4, 10.5)	34 (7.8%), 37	(5.5, 10.7)
Vomiting	14 (9.7%), 15	(5.4, 15.8)	14 (9.6%), 14	(5.3, 15.6)	15 (10.3%), 16	(5.9, 16.4)	43 (9.9%), 45	(7.2, 13.1)
Decreased appetite	44 (30.6%), 72	(23.2, 38.8)	53 (36.3%), 76	(28.5, 44.7)	44 (30.1%), 58	(22.8, 38.3)	141 (32.3%), 206	(28.0, 37.0)
Decreased activity level	66 (45.8%), 104	(37.5, 54.3)	62 (42.5%), 100	(34.3, 50.9)	55 (37.7%), 72	(29.8, 46.1)	183 (42.0%), 276	(37.3, 46.8)
Irritability	109 (75.7%), 209	(67.9, 82.4)	105 (71.9%), 201	(63.9, 79.0)	101 (69.2%), 145	(61.0, 76.5)	315 (72.2%), 555	(67.8, 76.4)

n (%), E: n = Count of Participants (at least one event i.e. Participants counted only once if the Participant reported one or more Events), % = (n / Number of Participants in Safety Population who received Dose 1 of Investigational Product)*100, E = Count of Events (Participant may be counted more than once).

At least one unsolicited AE was observed in 64.4% (95% CI: 59.6, 68.9) of the study participants (Table 4). The most frequently reported unsolicited events were the diseases commonly reported in the neonatal period including upper respiratory tract infection, respiratory tract infection, diarrhea, rhinitis, and conjunctivitis, with comparable distribution patterns between groups. All the unsolicited events were mild to moderate in intensity except for 6 severe AEs. All except three events (two events of pyrexia with one each in ROTAVAC and ROTARIX groups and one event of diarrhea in ROTAVAC 5D group) were assessed to be not related to the study vaccines. A total of 6 SAEs were reported in the study and included three events of bronchiolitis, two of sepsis, and one of diarrhea. Of these, only one event of diarrhea in the ROTAVAC 5D group was considered related to the vaccine as this outcome has been described previously to be associated with oral rotavirus vaccines, and the onset was within 7 days of vaccination. No death or case of intussusception was reported during the study period.

**Table 4:** Summary of Unsolicited Adverse Events - All Doses Combined – Safety Population.

Category of unsolicited AE	Rotavac (N = 139)		Rotavac 5D (N = 137)		Rotarix (N = 142)		Total (N = 418)	
	n (%), E	95% CI	n (%), E	95% CI	n (%), E	95% CI	n (%), E	95% CI
At least one unsolicited AE	95 (68.3%), 156	(59.9, 76.0)	93 (67.9%), 180	(59.4, 75.6)	81 (57.0%), 116	(48.5, 65.3)	269 (64.4%), 452	(59.6, 68.9
At least one related unsolicited AE	0	(0.0, 2.6)	2 (1.5%), 2	(0.2, 5.2)	1 (0.7%), 1	(0.0, 3.9)	3 (0.7%), 3	(0.1, 2.1)
At least one SAE	1 (0.7%), 1	(0.0, 3.9)	2 (1.5%), 2	(0.2, 5.2)	3 (2.1%), 3	(0.4, 6.0)	6 (1.4%), 6	(0.5, 3.1)
At least one related SAE	0	(0.0, 2.6)	1 (0.7%), 1	(0.0, 4.0)	0	(0.0, 2.6)	1 (0.2%), 1	(0.0, 1.3)
At least one unsolicited AE leading to withdrawal from study	0	(0.0, 2.6)	0	(0.0, 2.7)	0	(0.0, 2.6)	0	(0.0, 0.9)
At least one unsolicited AE leading to withdraws from study vaccination but remaining in the study	0	(0.0, 2.6)	0	(0.0, 2.7)	0	(0.0, 2.6)	0	(0.0, 0.9)
At least one unsolicited AE leading to hospitalization	1 (0.7%), 1	(0.0, 3.9)	2 (1.5%), 2	(0.2, 5.2)	3 (2.1%), 3	(0.4, 6.0)	6 (1.4%), 6	(0.5, 3.1)
At least one unsolicited AE leading to death	0	(0.0, 2.6)	0	(0.0, 2.7)	0	(0.0, 2.6)	0	(0.0, 0.9)
n (%), E: n = Count of Participants (at least one event i.e. Participants counted only once if the Participant reported one or more Events), % = (n / Number of Participants in Safety Population who received Dose 1 of Investigational Product)*100, E = Count of Events (Participant may be counted more than once)								

## 5 Discussion

In this Phase 2b study, we compared the immune responses of the WHO prequalified formulation of ROTAVAC, which is formulated as a frozen product, with those of ROTAVAC 5D, a more heat-stable formulation. This is also the first clinical assessment of the two vaccines outside of India, thus constituting critical data to support country-level decision-making on rotavirus vaccine product choice across multiple geographies. We found that both ROTAVAC 5D and ROTAVAC were immunogenic and safe in this population and performed equally well, as non-inferiority in terms of immunogenicity of one vaccine over the other was demonstrated.

To ensure that the ROTAVAC results can be directly compared with the earlier studies conducted in India we used the anti-rotavirus IgA assay that employs WC3 viral lysate as the antigen at the same laboratory where pre-licensure testing was conducted. Post-vaccination GMCs reported from India were around 20 U/ML with 4-fold seroresponse rates between 29.2%% to 38.6% [6], [7], while in this study we report GMCs of 18.1 and 14.0 and 4-fold seroresponse rate of 33.6% and 27.4% for ROTAVAC 5D and ROTAVAC, respectively. These similarities support the reliability of the assay as well as the consistent performance of the vaccines despite the ethnic, geographic, and regional differences in the recipients.

Our approach to determine non-inferiority of ROTAVAC 5D with respect to ROTAVAC applied robust statistical methodology by establishing an *a priori* threshold GMC ratio between the two vaccines of > 0.5 to demonstrate immunologic non-inferiority of the former to the latter. These criteria have been used previously in comparing other rotavirus formulations [15].

In the Phase III efficacy trial of ROTAVAC conducted in India, the efficacy of ROTAVAC against severe rotavirus gastroenteritis was 56.3% during the first year of life, 48.9% in the second year of life, and 55.1% overall up to two years of age [4], [5]. The efficacy study also reported a 4-fold increase above baseline in serum anti-rotavirus IgA in 39·9% of the vaccine recipient. The similarity in the immune response seen in this study with that observed in the Phase III clinical studies provides some indication that comparable protection may be expected across different geographies.

The selection of the WC3 rotavirus strain in the primary assay to compare ROTAVAC and ROTAVAC 5D was based on the large body of experience gained from its use in the clinical development of ROTAVAC. Although WC3 is heterologous with respect to all the vaccines tested, including strain 116E (the base strain for ROTAVAC), the serum IgA assay was fully validated with that strain and was used throughout the clinical development that led to the licensure and WHO prequalification of ROTAVAC. Use of this test to make comparisons with ROTARIX immunogenicity could be inappropriate, as WC3 is also heterologous with respect to 89–12, the base strain from which ROTARIX was developed. However, in order to obtain an indication of the performance of ROTARIX in the study, we tested a subgroup of infants from each group using 89–12 antigen in the assay. While we were able to confirm the immunogenicity of ROTARIX, we avoided making comparisons between ROTARIX and ROTAVAC. A comparison of the immune responses for ROTAVAC vaccines using ELISA with WC3 assay and ROTARIX using ELISA with 89–12 strain was also not conducted as it is well known that ELISAs using homologous strains show higher titres when compared with titre from ELISA using heterologous strain and WC3, considered heterologous strain for 116E strain in rotavirus vaccine was expected to show lower titres [10], [16].

ROTAVAC and ROTAVAC 5D were generally well tolerated with no noted differences in the rate of solicited events, unsolicited AEs, or SAEs observed between the two groups.

A majority (almost 88%) of the participants reported a mild-to-moderate solicited AE with most resolving within seven days of vaccination. It is worth noting that all participants received routine infant vaccines concomitantly, including DTwP-HepB-Hib, which is associated with high rates of fever following vaccination.

Based on our results, we conclude that ROTAVAC 5D is immunologically non-inferior to ROTAVAC when administered orally along with other routine vaccines in Zambian infants. ROTAVAC 5D induces humoral responses similar to those observed during the ROTAVAC trial conducted in India, wherein clinical efficacy was also demonstrated. Additionally, both ROTAVAC formulations had an acceptable safety profile and were well tolerated when incorporated into the routine infant immunization schedule in Zambia. These data should provide reassurance to immunization policy-makers worldwide that these vaccines will perform similarly in their own local contexts, thus allowing countries to have greater flexibility when considering different rotavirus vaccine options.

## Declaration of Competing Interest

The authors declare that they have no known competing financial interests or personal relationships that could have appeared to influence the work reported in this paper.
