# Supplementary material for: Immunogenicity and safety of two monovalent rotavirus vaccines, ROTAVAC® and ROTAVAC 5D® in Zambian infants
Source: Vaccine. 2021 Jun 16;39(27):3633–40. doi: 10.1016/j.vaccine.2021.04.060 (PMC8204902; doi:10.1016/j.vaccine.2021.04.060)
Supplement: Supplementary data 1 [file mmc1.docx]

**Supplementary Tables**

**Supplementary Table 1: Additional Post- Vaccination Immune Responses for ROTAVAC and ROTAVAC 5D, in terms of Serum Anti-Rotavirus IgA Antibody Concentrations Measured by ELISA using WC3 strain as viral Lysate– PP Population**

|  | **ROTAVAC** | | | **ROTAVAC 5D** | | | **Comparison** |
| --- | --- | --- | --- | --- | --- | --- | --- |
|  | **N** | **n** | **GMC/ % / GMFR (95% CI)** | **N** | **n** | **GMC/ % / GMFR (95% CI)** | **GMC Ratio (ROTAVAC 5D/ ROTAVAC)/ % difference / GMFR Ratio (Post-vaccination/ Pre-vaccination) (95% CI)** |
| Seroresponse rate- Two-fold rise | 124 | 41 | 33.1 (24.9, 42.1) | 128 | 52 | 40.6 (32.0, 49.7) | 7.6 (-4.4, 19.3) |
| Seroresponse rate- Three-fold rise | 124 | 40 | 32.3 (24.1, 41.2) | 128 | 49 | 38.3 (29.8, 47.3) | 6.0 (-5.8, 17.7) |
| Definitions:  Seroconversion: Post-vaccination serum anti-rotavirus antibody IgA concentration of at least 20 U/mL if a baseline concentration was < 20 U/mL, or ≥ 2-fold baseline level if the baseline concentration was ≥ 20 U/mL.  Seroresponse in terms of a n-fold rise: Post-vaccination serum anti-rotavirus antibody IgA concentration of ≥ maximum of 20 U/mL and n-fold LLOQ if the baseline concentration was ≤ LLOQ, or ≥ maximum of 20 U/mL and n-fold baseline level if the baseline concentration was > LLOQ. | | | | | | | |
